# Closest horizons of Hsp70 engagement to manage neurodegeneration

**DOI:** 10.3389/fnmol.2023.1230436

**Published:** 2023-09-19

**Authors:** Artem A. Venediktov, Olga Yu Bushueva, Varvara A. Kudryavtseva, Egor A. Kuzmin, Aleksandra V. Moiseeva, Anna Baldycheva, Igor Meglinski, Gennadii A. Piavchenko

**Affiliations:** ^1^Department of Human Anatomy and Histology, I.M. Sechenov First Moscow State Medical University (Sechenov University), Moscow, Russia; ^2^Laboratory of Genomic Research, Research Institute for Genetic and Molecular Epidemiology, Kursk State Medical University, Kursk, Russia; ^3^STEMM Laboratory, University of Exeter, Exeter, United Kingdom; ^4^Department of Physics, University of Oulu, Oulu, Finland; ^5^College of Engineering and Physical Sciences, Aston University, Birmingham, United Kingdom

**Keywords:** Hsp70, neuropharmacology, Alzheimer’s disease, amyotrophic lateral sclerosis, neurodegenerative diseases

## Abstract

Our review seeks to elucidate the current state-of-the-art in studies of 70-kilodalton-weighed heat shock proteins (Hsp70) in neurodegenerative diseases (NDs). The family has already been shown to play a crucial role in pathological aggregation for a wide spectrum of brain pathologies. However, a slender boundary between a big body of fundamental data and its implementation has only recently been crossed. Currently, we are witnessing an anticipated advancement in the domain with dozens of studies published every month. In this review, we briefly summarize scattered results regarding the role of Hsp70 in the most common NDs including Alzheimer’s disease (AD), Parkinson’s disease (PD), and amyotrophic lateral sclerosis (ALS). We also bridge translational studies and clinical trials to portray the output for medical practice. Available options to regulate Hsp70 activity in NDs are outlined, too.

## 1. Introduction

Although neurodegenerative diseases (NDs) are rather widespread while their course is severe and prone to progression with increasing cognitive dysfunction and fatal outcomes, we still possess no effective tools to achieve a critical improvement in incidence and mortality. The problem is not only medical and not uniquely longevity is affected. Since senescence is actually a principal risk factor (Turturici et al., 2011), NDs impair person’s ability, shrinking working life. As the clinical and socioeconomic impacts remain relevant, studies of pathogenetic mechanisms in neurodegeneration are needed to develop novel approaches for its detection and treatment. The most important pathogenic links of NDs involve oxidative stress, mitochondrial dysfunction, neuroinflammation, excitotoxicity, and defects of autophagy. Finally, the hallmark is accumulation of protein aggregate deposits, reflecting critical imbalance in neuronal homeostasis.

In neurodegenerative brain, the cells tend to synthesize misfolded proteins and lose an ability to properly utilize them (Campanella et al., 2018). Because neurons form sophistic anatomically and functionally interconnected networks, each cell may serve a crucial link in numerous different circuits. Thus, for neurons it is dramatically important to stay alive as long as possible, and this is one of the reasons for the longest neuronal lifetime (Kole et al., 2013). The last feature, together with highest complexity and diversity of brain’s proteome (Mauger and Scheiffele, 2017; Negi and Guda, 2017; Korovesi et al., 2020; Sinitcyn et al., 2023) determines a need for very thorough quality control of cerebral polypeptides.

Maintaining healthy proteome requires control over newly synthesized proteins as well as clearance/cleavage or refolding of unstable mature ones. All these functions are naturally provided by highly conservative molecular machinery called chaperones. Among the others, the family of heat shock proteins with a molecular weight of 70 kDa (Hsp70) is considered the most important in terms of neurological disorders. Hsp70 represent one of the chief groups among the protectors, being phylogenetically very old and preserved (Koren et al., 2009). These facts invite us to study the potential pharmacological benefit of Hsp70 in NDs.

Numerous studies have revealed the contribution of Hsp70 in degenerative and age-associated anomalies of the brain. However, some of the positive roles of Hsp70 in brain pathology remain difficult to estimate in terms of its significance for translational medicine. Herein, we summarize the state-of-the-art and previous advances disclosing the clinical potential of the strategies based on Hsp70 management. Especially important focus in this review is given to clinical trials, which bring Hsp70 closer to the nearest practical application.

## 2. Essentials of Hsp70

### 2.1. Hsp70 family

The family of Hsp70 includes more than 10 members (Table 1). Generally, they enable adequate folding for both newly synthesized or mature proteins as well as refolding for denaturated/aggregated proteins (Hartl and Hayer-Hartl, 2002). In addition, some constitutively expressed or induced members of the family possess relatively specific functions, such as regulation of apoptosis, mitochondrial function (mtHsp70), and metabolic pathways (Lackie et al., 2017). However, Hsp70 are specified to ensure folding *via* ATP-dependent machinery, preventing denatured proteins from aggregating (Jores et al., 2018).

**Table 1:** Key Hsp70 ambassadors.

HSP70 member and its alias	Typical localization	Features and arguable facts	References
Hsp70, or heat shock protein proper, or Hsp72, or HspA1	Mainly cytoplasm; nucleus, plasma membrane	Reveals chaperone properties; its expression is principally induced by stress stimuli like hyperthermia, oxidation, and hypoxia (HspA1A is the most common version, while HspA1B and HspA1L homologs almost do not differ); a recruitment to the plasma membrane is mediated *via* phosphatidylinositol	Turturici et al. (2011), Radons et al. (2016), and Smulders et al. (2022)
HspA2	Extracellular vesicles	No obvious found action; the levels are increased in proteotoxicity	Sojka et al. (2023)
HspA3	Cytosol	Assumed as not fulfilling Hsp70 definition	Gabriele et al. (1996)
HspA4 and HspA4L, or Grp110	Cytosol	Selection of anti-apoptotic options in the relevant cascades; sometimes is regarded as Hsp110	Kaneko et al. (1997)
Grp78, or HspA5	Endoplasmic reticulum; plasma membrane	Present in any normal growth, appear inside extracellular exosomes; contribution to the development of ER-associated apoptotic infrastructure; sometimes may be found on cell membranes but with no steady attachment	Zhang et al. (2010), Turturici et al. (2011), and Radons et al. (2016)
HspA6	Cytosol, may be extracellular	Enhances proliferation *via* Salvador-Warts-Hippo metabolic pathway	Zhang L. et al. (2023)
HspA7	Cytosol, may be extracellular	Takes part in oncogenesis by an unclear machinery, although acting like a molecular pattern for toll-like receptors, TLR-2 especially	Feng et al. (2022)
Hsc70 (heat shock protein cognate), or Hsp73, or HspA8, or HspA10	Cytosol (basic cytosol form), traces in the nuclei	Chaperoning, ubiquitination, aggregate prevention, normal cellular functioning; a keystone of CMA in Hsp70 (sometimes in modifications)	Turturici et al. (2011), Lizama et al. (2018), Rai et al. (2021), and Rai and Tapadia (2022)
lysHsc70	Lysosomes	Lysosomal modification of the cytosolic isoform; binds KFERQ-patterns in polypeptide chains recognizing them as degrons	Ciechanover and Kwon (2017)
Grp75, or HspA9, or MtHsp70, or mortalin	Mitochondria, nucleus	Present in any normal growth; binds p53 preventing its antioncogenic shield; enables protein transfer through mitochondrial membranes (crucial for Bcl-2-based transfer of glucocorticoid receptors)	Mizukoshi et al. (1999), Wadhwa et al. (2002), and Havalová et al. (2021)
HspA11	No data applicable	No data applicable	Few evidence, which are not serious enough to consider
HspA12 subfamily: HspA12a and HspA12b mainly	Cytoskeletal structures, cytosol, exosomes	Principally found in brutal environmental fluctuations like humidity, temperature, etc.; a precise machinery is not completely described in mammals or other chordates	Hu et al. (2019) and Clark et al. (2021)
HspA13	Endoplasmic reticulum and cytosol	Recently exposed to a comprehensive examination; seems to control a proper folding of normal nascent proteins	Espinoza et al. (2022)
HspA14	Nucleus and cytoplasm	Most likely, inhibits viral genome transcription in retroviruses and thereby constitutes a frontline of non-immune anti-HIV-struggle	Bi et al. (2023)
SecHsp70: usually HspA1 of any of three genes coding	Extracellular matrix	Defense against toxic action of protein aggregates outside cells; engineered form or pathological release with an outlined inflammatory phenotype	De Mena et al. (2017)

The list discloses cornerstone actors of the family of 70 kDa-weighed Hsp; five of them are the most common in cells and thereby interesting (HspA1, Hsc70, mtHsp70, Grp78, and lysHsc70) while secHsp70, that is an extracellular HspA1 alias, displays an ambivalent role.

**Figure 1:**
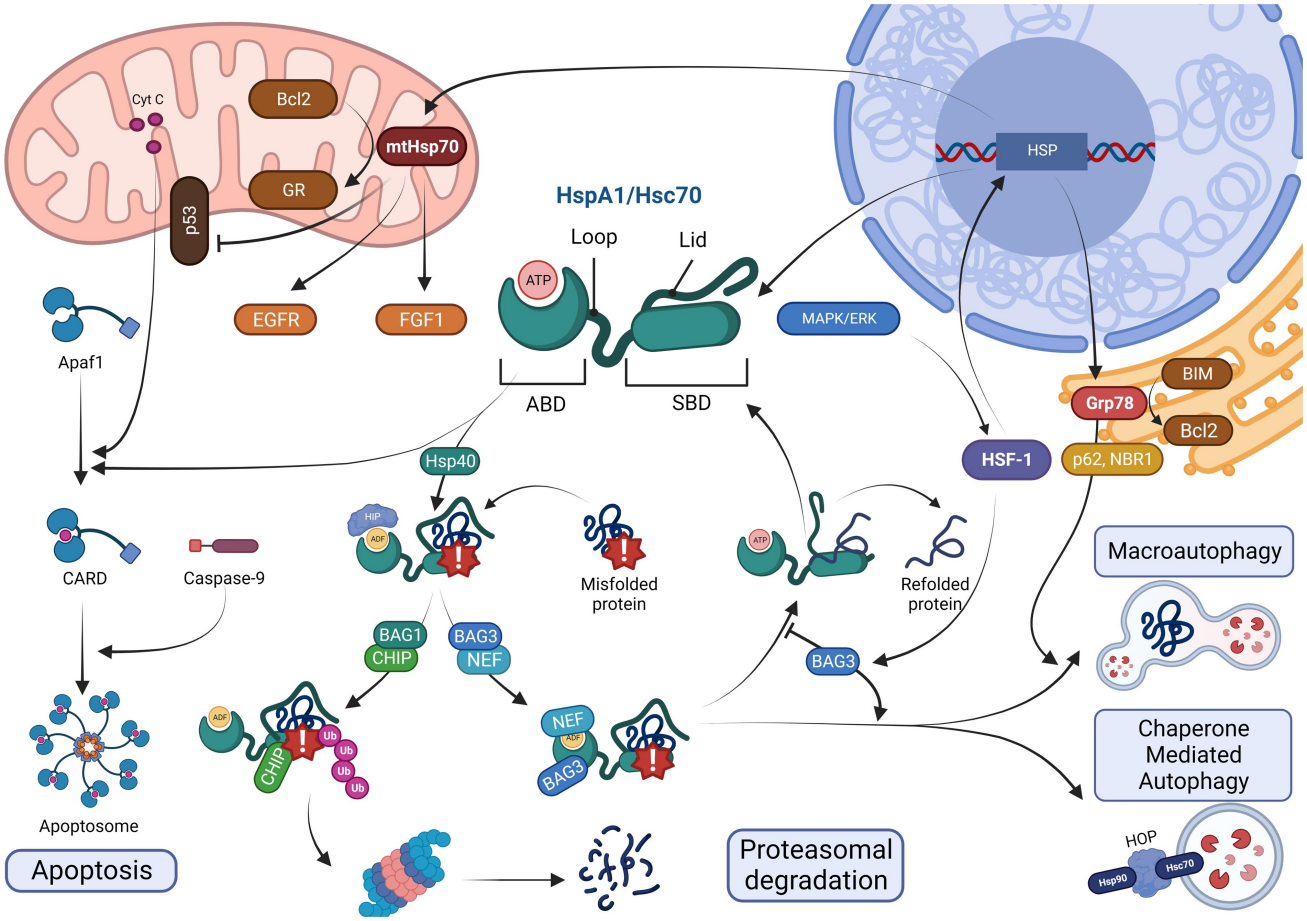
Toggles between crucial options: a model of intracellular pathways for common Hsp70 members (HspA1, Hsc70, Grp78, and mtHsp70) at their impact sites. At the center, one can see HspA1 and Hsc70, which are the two most prevalent players in the cytosol, with their active structures comprising four structures in large: an ABD (at the N-terminus) to engine chaperone activity, a loop (fine and folded linker) to contact many allosteric regulators, an SBD (beta-barrel) to capture misfolded proteins, and a lid (motile cap-like chain) to either come closer to the SBD or to align depending on ATP-ADP dualism. When HSF-1-upregulated transcription is completed, the cytosolic isoforms enter Fork # 1, where Hsp40 navigate a grip of a hazardous protein (down) *via* dephosphorylation and ADP inactivation by HIP, whereas the lid closes over the problematic chain; otherwise, apoptotic recruitment may occur (left). In apoptosis, cytochrome c-induced APAF1-driven recruitment of caspase-9 into the CARD is blocked by Hsp70. In contrast, the Hsp40-switched track comes into its Fork # 2 with a BAG1-associated choice of an allosteric CHIP regulation towards ubiquitin-mediated proteasomal degradation (below) or a BAG3-associated concurrent NEF-inhibition of the ABD up to Fork # 3 (right) with a continuous HSF-1 presence leading to either BAG3-overexpression and autophagic events or holding of the hazardous substance inside the beta-barrel until the BAG3 content decreases with NEF released and protein returned into the cytosol to refold in better conditions (up). Autophagic Fork # 4 occurs either on the ER surface for Grp78 modification of Hsp70 (on the right) driven into macroautophagy by p62 and NBR-1 or in the cytosol with Hsp90-recruiting HOP assistance, causing chaperone-mediated autophagy, which is more typical for Hsc70 immersion into lysosomes. Grp78 also acts against apoptosis, preventing BIM from Bcl2 elimination. In the left upper corner, mtHsp70 is found; it is anti-apoptotic, too, *via* an induction of Bcl-2 to transfer GR into the organelles. A positive impact on EGFR and FGF-1 activity as well as a negative impact on p53 chains is clear.

Heat shock proteins are found in almost all cellular compartments, including the nucleus and cytoplasm (Hsc70), as well as in mitochondria (Wentink et al., 2020). Moreover, Hsc70 may be found in a lysosome-specific isoform (Ciechanover and Kwon, 2017). General protective properties of Hsp70 determine both pro- and eukaryotic biology, and even plants express their own Hsp70 analogs (Chaudhary et al., 2019). However, some features are shared by principal chaperones of the group for mammals, including humans, mice, and rats (Figure 1). Hsc70, which is the most common one among the Hsp70 in healthy and undamaged state, has the largest number of unique interacting proteins among all the Hsp70 family members (Rai et al., 2021).

### 2.2. Cellular regulation of Hsp70

Hsp70 expression is controlled by heat shock factor 1 (HSF-1), which receives downstream signals from a number of stimuli, such as pathway of extracellular signal-regulated kinases and mitogen-activated protein-kinases (ERK/MAPK), highly influenced by increased air pressure and temperature (Matsathit et al., 2016). HSF-1 is also a thermosensor whose leucin zipper domains change in temperature bounces. Moreover, Hsp70-induced feedback permits to monomerize trimers of HSF-1 and separate it from DNA molecules (Kmiecik and Mayer, 2022).

The activity of Hsp70 is driven by small Hsp with a molecular weight of 40 kDa (Hsp40/DnaJ family), especially DnaJB11 and DnaJC5 (Braun, 2023), which are relevant for some types of tauopathies (Kampinga and Craig, 2010; Zhang et al., 2023, M). Although the activity of Hsp70 is firstly dependent on its expression, some enzymes also may introduce modifications, regulating Hsp70 in kinase dependent manner. For instance, it has been reported that cyclin-dependent kinase 1 (Cdk1) is able to temporarily downregulate chaperonic functions of Hsp70 by phosphorylation of serine in a region between ATP- and substrate-binding domains (Kao et al., 2020). Interestingly, in addition to *de novo* synthesis of Hsp70, neurons tend to uptake it from neighboring astrocytes (Tytell et al., 1986; Hightower and Guidon, 1989; Guzhova et al., 1998, 2001; Kalmar and Greensmith, 2017).

## 3. Hsp70 in NDs

### 3.1. Hsp70 and aging

Hsp70 play an important role in the nervous system in health and in disease. It is especially remarkable for aging brain. The aging itself is a crucial risk factor as any senescent cell exhibits a lower chaperone protein translation while its markers for labeling of misfolded proteins are not expressed well (Llewellyn et al., 2023). For instance, Hsp70 production falls by 50% in aged rat liver cells in stress (Heydari et al., 1994). Basal Hsp70 content in cells stays higher in long-living animals (de Toda et al., 2016). Furthermore, co-chaperones of Hsp70 in mice are expressed less in development of age-dependent neurodegeneration (Lackie et al., 2020). Higher levels of oxidant and lower ones of antioxidant actors may aggravate the state of chaperone action in aged individuals (Martínez de Toda and De la Fuente, 2015).

Despite these facts, not all the heat shock proteins decrease at the same rate as Hsp70 do in aging. Specifically, ATP-recruiting Hsp (foldases) experience a strong hypoexpression, while ATP-independent (holdases) continue to be actively synthesized (de Graff et al., 2020). As a result, proteostasis and preventing of aggregation suffers less than the protection of nascent proteins. Sirtuins, which are well-known for their controlling role in aging regulation, are probably responsible for proper Hsp70 expression after the stimulation by HSF-1. Common in young age, sirtuin 1 has been evidenced to potentiate binding of HSF-1 to DNA, thereby modifying levels of Hsp70 synthesis (Karvinen et al., 2016).

### 3.2. Hsp70 in Alzheimer’s disease

Alzheimer’s disease (AD), known as the most widespread ND, mainly affects memory and other cognitive functions related to the synaptic loss and deposition of neurofibrillary tangles (NFT) and peptide plaques (Lane et al., 2018). The plaques are principally formed by amyloid beta (Aβ; Abeta) deriving from Aβ precursor (APP) after enzyme-mediated cleavage, especially under presenilin-1 (PS1; PSEN1) action (Russo et al., 2000; Rajesh and Kanneganti, 2022). Despite being debatable, this Aβ hypothesis of AD is still considered to be generally accepted (Breijyeh and Karaman, 2020). Recent studies also claim soluble Aβ oligomers (AβOs) to have more harmful impact than their insoluble counterparts. For instance, excitotoxicity and abnormal long-term potentiation are already seen at very early stages of AD before detectable plaques (Huang and Liu, 2020). In fact, multifaceted pathways of AD imply numerous links described in detail in excellent reviews (Perrin et al., 2009).

Elevated levels of Hsp70 are found in AD, whereas the chaperones obviously attenuate the disease (Romi et al., 2011). For example, protein aggregation and neuronal death together with AD-like symptoms, caused by the use of paraquat, a popular herbicide in many countries, are accompanied by a reduced Hsp70 expression (Moyano et al., 2021). On the other hand, an upregulation of Hsp70 has correlated with a decline in Aβ content (Sun et al., 2017).

Excessive endogenous Hsp70 increases the efficiency of Aβ-degrading enzymes (Hoshino et al., 2011). Rivera and coll. Have observed Hsp70 affecting the Aβ assembling process *in vitro* preventing oligomer formation. Moreover, the presence of Hsp70 reduced the Aβ peptide-induced toxicity of cultured neurons (Rivera et al., 2018). In drosophila genetic models, hyper-expressing secreted Hsp70 (secHsp70) together with mutant Aβ42, reveal that the extracellular Hsp70 only provided a protective action (De Mena et al., 2017). Some studies have found a similar pattern of Hsp70 impacts for both localizations. Thus, the potential for cognitive protection remained the same for both extra- and intracellular Hsp70 for drosophilae with Aβ accumulation regardless of their age or exposure (Martín-Peña et al., 2018).

Cytoskeletal instability as a result of the production of impaired tau protein is another important component of AD pathology (Choi et al., 2020). It is generally accepted that an increased activity of tau kinases, especially of glycogen-synthase kinase 3 (GSK-3), extracellular signal-regulated kinase (ERK), and p38, leads to the hyperphosphorylation of tau (Hartz et al., 2023). As a result, tau molecules aggregate into double threads forming a net of NFT, that further intensifies the Aβ accumulation (Uematsu et al., 2018). It is shown that valosin-containing protein (p97; VCP) can eliminate the NFT with the help of Hsp70 (Saha et al., 2023). Overexpressed Hsp70 enables degradation or dephosphorylation of pathological tau improving stability of microtubules (Lackie et al., 2017).

Despite all the positive effects, at the late stages of cellular pathology Hsp70 loses its protective functions and forms epichaperomes representing inert long-living scaffolds (Bolaender et al., 2021). This phenomenon enables protein connectivity-based dysfunction and might aggravate the neurodegeneration (Inda et al., 2020). Additionally, Aβ and tau suppressive activity of Hsp70 has been shown to be abolished in the presence of ε4 isoform of apolipoprotein E (ApoE), one of the most recognizable factors contributing to AD (Osorio et al., 2007; Serrano-Pozo et al., 2021). Finally, although the subchronic intranasal administration of human Hsp70 has improved the course of disease in familial AD murine models, innate immunity and antigen presentation have been upregulated at the same time (Evgen’ev et al., 2019).

### 3.3. Hsp70 in Parkinson’s disease

Parkinson’s disease (PD) is a common neurodegeneration, clinically displayed in bradykinesia, postural instability, rigidity, and tremor (Ascherio and Schwarzschild, 2016). The disorder is caused by a progressive death of dopaminergic neurons in the substantia nigra (Beal, 2010). The hallmark of the disease required to confirm the diagnosis is intracellular inclusions called Lewy bodies (LB) (Spillantini et al., 1997; Sipilä et al., 2023). LB are composed of ubiquitinated abnormal protein called α-synuclein (α-Syn) (George, 2002). In addition to α-Syn, some other causative players have been shown to contribute to the disease: Parkin, encoded by PARK2 gene; phosphatase and tensin homolog-induced kinase 1, encoded by PINK1 or PARK6; protein deglycase J-1 (DJ-1), encoded by PARK7; and leucine-rich repeat kinase 2 (LRRK2), encoded by PARK8 (Poewe et al., 2017).

In PD, Hsp70 overexpression is common in damaged cells, generally in surviving neurons (Dickson, 2018; Jellinger, 2019). Consistently, Drosophila models have disclosed Hsp70 to delay death of dopaminergic cells (Auluck and Bonini, 2002). On one hand, the benefit of Hsp70 might be explained by its ability to enhance autophagy (Moors et al., 2017; McKinnon et al., 2020). Nonetheless, mitophagy and elimination of Parkin also require HspA1 presence, and of HspA1A mainly, as HspA1L potential is weaker (Hasson et al., 2013). However, Hsp70 machinery in PD is not acting alone, but strongly depends on Hsp40, Hsp90, and Hsp70-Hsp90-organizing protein (HOP) (Ebrahimi-Fakhari et al., 2012; Wu et al., 2019; Hu et al., 2021). Moreover, small molecular chaperones, e. g. clusterin (ApoJ), are probably more prominent to manage α-Syn aggregation in PD (Lenzi et al., 2020).

### 3.4. Hsp70 in Huntington’s disease

Huntington’s disease (HD) is a complex motor (choreal signs are rather common), cognitive, and mental neurodegenerative disorder, which is inherited *via* an autosomal dominant pattern (Joshi et al., 2021). HD is the most common representative of a wide range of polyglutamine-associated diseases (polyQ), including also spinocerebellar ataxias and dentatorubral-pallidoluysian atrophy (Carroll et al., 2018). All of them imply a translation of polyQ-chains forming Htt protein after posttranslational modification (Bates et al., 2015). Htt aggregates in essentially all neuronal and astrocytic compartments (Joshi et al., 2021; Lange et al., 2023).

Hsp70 may promote the collapse of polyQ chains (Choudhury et al., 2016; Davis et al., 2020; Gupta et al., 2020). In insects, Hsc70 is responsible for the effect (Rai and Tapadia, 2022). However, there is a lack of data supporting active Hsp70 engagement in the treatment of these disorders in mammals (Pratt et al., 2015; Reis et al., 2017). Maheshwari and coll. Have tried steroid hormones to manage Hsp70 machinery in HD, but no specific action is shown (Maheshwari et al., 2014). Some studies propose an anti-apoptotic role of Hsp70 in HD (Sabirzhanov et al., 2012). Besides, an indirect explanation of Hsp70 relevance in HD is given by the fact that progenitor nerve cells express more Hsp40 (co-chaperone of Hsp70) than mature neurons, whereas polyQ-associated disorders usually manifest in adult persons and not in childhood (Thiruvalluvan et al., 2020). Perhaps, the Hsp40 is the key factor to trigger polyQ removal.

### 3.5. Hsp70 in amyotrophic lateral sclerosis and frontotemporal dementia

Amyotrophic lateral sclerosis (ALS) is a progressive motor neuronal disorder (Amico and Antel, 1981). Despite a long history of studies, the interactions between its key actors are still not completely clear (Al-Chalabi et al., 2017). Superoxide dismutase 1 (SOD-1) is often considered to be crucial in pathology (Cleveland, 1999). However, products of fused-in-sarcoma (FUS) gene are important contributors to protein aggregation, and such faulty proteins as transactive response DNA binding protein 43 kDa (TDP-43), C9orf72, and ubiquilin-2 are also involved (Thomas et al., 2013). Moreover, there is a second row of proteins, which are able to aggravate the course of the disorder. So, NF-kappa-B activator-binding kinase 1 of TNF receptor-associated factor’s family member (TBK1), optineurin, and p62 participate in impaired autophagy, while vesicle-associated membrane protein-associated protein B/C (VAP-B) is typical for proteasomal failure. Although, VCP takes part in both machineries (Kalmar and Greensmith, 2017).

ALS and frontotemporal dementia (FTD) may be regarded together because of similar genomics, proteomics, metabolomics, and transcriptomics (Menéndez-González, 2023). Mandrioli et al. (2019) also mention that both diseases are accompanied by neuroinflammation with inflammasomes. FTD is a group of similar threatening and rapidly progressing cognitive disorders with a rather high mortality (Wang et al., 2022). The pathology comprises a storage of pathological tau, Fus, and TDP-43 with predominant harm to the neurons in the frontal and temporal cortex (Josephs et al., 2011). Furthermore, repeated sequences of G4C2 in C9orf72 genes are notorious for the synthesis of dipeptide repeat proteins (DPR) or poly-Gly-Ala (poly-GA), tending to aggregate (Lee et al., 2023). Interestingly, DPR complexes are able to be transported to neighboring cells, thereby hindering their clearance (Khosravi et al., 2020).

Hsp70 is found to assist the elimination of the abnormal proteins, especially in the DPR-related pathology (Deng et al., 2011). Generally, in ALS and FTD Hsp70 act *via* either autophagy to prevent aggregation of RNA-binding proteins (Mandrioli et al., 2019) or proteasomal machinery to fix DPR-induced damage. In proteasomes, ubiquilin-2 provides a rapid Hsp70 binding to poly-GA (Renaud et al., 2019). The intermediate region of ubiquilin-2 molecule includes a flexible proline-X-X-proline (PXXP) motif, which is perhaps accessible for concurrent catalysis or inhibition (Zhang et al., 2021). Meanwhile, these RNA-binding proteins comprise TDP-43 and Fus, and may be cleaved by such kinds of autophagy as chaperone-mediated autophagy and aggrephagy (Thomas et al., 2013). Aggrephagy implies a consumption of labeled stress granules from aberrant RNA (Ripin and Parker, 2022). Interestingly, TDP-43 and ubiquilin-2 seem to be tightly interacting despite their different roles in proteasomal machinery and autophagy (Seelaar et al., 2007; Wang et al., 2008).

## 4. Hsp70 as a target to treat neurodegeneration

Current drug options in NDs probably lack Hsp70 usage to improve neuronal protein quality control (Table 2). Some medications, such as BGP-15 (*O*-[3-piperidino-2-hydroxy-1-propyl]-nicotinic acid amidoxime dihydrochloride) and celastrol, have not shown a real positive Hsp70-mediated impact on neurodegeneration (Kalmar and Greensmith, 2017). New candidates should pass by a number of studies, and *in vitro* ATP-ase tests are preferred initially (Repalli and Meruelo, 2015). Surprisingly, in some cases Hsp70-related drugs have even aggravated NDs. Johnson and coll. Emphasize a positive correlation between spinocerebellar ataxia and elevated Hsc70 content in a Drosophila model (Johnson et al., 2020), though it may be explained either as a harmful phenomenon or cell resistance to the pathology.

**Table 2:** Core set of Hsp70-mediated medications in NDDs.

Factor	Hsp70	Scenario	References
Arimoclomol	Induction	Downregulation of glycosphingolipid storage; prolongation of HSF-1 activation with a stress-resistance maintained	Kalmar et al. (2014), Kirkegaard et al. (2016), and Kalmar and Greensmith (2017)
Cannabidiol	Induction	Grp78 overexpression with a reversal of the apoptotic watershed in ER	Patel et al. (2023)
Carbenoxolone	Induction	HSF-1 activation in rotenone presence	Thakur and Nehru (2014)
Colchicine	Induction	Upregulation of HspB8 expression and blocking TDP-43 accumulation	Mandrioli et al. (2019)
Fenofibrate	Induction	Abundant cytokine cascades with a synchronous Hsp90 decline and Hsp70 elevation	Rizk et al. (2022)
Phenlarmide	Induction	Hsp70-mediated α-synuclein disaggregation	Bao et al. (2017)
GGA/geranyl geranylacetone	Induction	Activation of ERK/p38 MAPK signaling pathway and retardation of inflammatory reactions	Sun et al. (2017)
HSF-1 proper	Induction	Upregulation of Hsp70 transcription; selective toxicity blocking with no trimerization or modification of signaling pathways	Kondo et al. (2013) and Verma et al. (2014)
J147	Inhibition	Prevention of synaptic protein loss and thus of cognitive dysfunction in a diminished Hsp70 expression; an overexpression of Hsp90	Chen et al. (2011)
Luteolin	Induction	Mechanism and causation are not clear yet	Rahimpour et al. (2023)
Myricetin	Induction	Proteasome-mediated cleavage	Joshi et al. (2019)
Neferine	Induction	Hsp70-mediated tolerance to hypoxia	Sengking et al. (2022)
Phenothiazines: methylene blue or leucomethylene blue dimesylate (TRx0237) and azure C	Inhibition	ATP-ase activity inhibition	Lo Cascio and Kayed (2018)
Pioglitazone	Induction	Induction of Hsp70 in the pancreas	Konturek et al. (2005)
Rhodacyanine-derived compounds: MKT-077, YM-01, YM-08, and JG-23	Inhibition	Binding allosteric Hsp70 regions to provide tight interaction with misfolded proteins with no return in ATP-binding state and no protein release into the cytosol	Abisambra et al. (2013)
Riluzole	Induction	Stimulation *via* HSF-1 dependent upregulation	Yang et al. (2008)
*Tetracarpidium conophorum*	Induction	Credible results with an unclear machinery	Tokunbo et al. (2023)
U-133	Induction	HSF-1 transcription enhanced	Ekimova et al. (2018)

The medications here include all novel options mentioned in PubMed-indexed publications for two recent years as well as any option cited at least twice for previous years.

### 4.1. Hsp70 enhancers in NDs

Heat shock factors are first possible tools to rule Hsp70 in NDs (Verma et al., 2014). So, HSF-1 takes part in numerous pathways, thereby being a key point of regulation (Kim et al., 2017). In particular, HSF-1 is trimerized to bind a sequence called the heat shock element in Hsp70 gene promotors (Kondo et al., 2013). However, an excessive production of HSF-1 in cerebellar neurons has decreased Htt-related toxicity in rats not *via* a direct Hsp70 upregulation, but *via* such coactor as BAG-3 (Verma et al., 2014). In Hsp70-knockout (ko) mice, increased HSF-1 levels have also improved the number of dopaminergic neurons in treatment by U-133, a compound derived from sea urchins (Ekimova et al., 2018). Independently of direct or indirect Hsp70 engagement, geranylgeranyl acetone (GGA; teprenone) mitigates neuronal damage *via* HSF-1 activation through the ERK/p38 MAPK pathway (Sun et al., 2017) (Table 3).

**Table 3:** Some recent noteworthy clinical trials of Hsp70-driven medications in NDDs.

Medication	Model for trial	Results	References
Arimoclomol	Adults diagnosed with probable/definite ALS	Controversial results; no data about benefits in finals with good intermediate endpoints passing as for Year 2016	NCT03491462; NCT00706147; NCT00244244 (Kalmar and Greensmith, 2017)
Arimoclomol	Patients of 2–18 years with NPC	Arimoclomol showed a 65% retardation of annual disease progression	NCT02612129 (Mengel et al., 2021)
Colchicine + riluzole	Adults diagnosed with probable/definite ALS	Ongoing	NCT03693781 (Mandrioli et al., 2019)
J147	Healthy subjects (Phase I only)	No results found yet to be posted	NCT03838185
Leucomethylene blue dimesylate	Adults with diagnosis of all cause dementia and probable AD	Results revealed no benefit of the medication to treat patients with mild to moderate AD	NCT01689246 (Gauthier et al., 2016)
Phenlarmide	Parkinson’s disease	No results found yet to be posted	NCT04693039; NCT04164121
Pioglitazone + riluzole	Adults diagnosed with probable/definite ALS	A clinical improvement was shown with no distinct role of Hsp70 elucidated although it is theorized	NCT00690118, NCT00919555 (Dupuis et al., 2012)
Riluzole	Adults diagnosed with probable/definite ALS	Riluzole 100 mg daily was found prone to improve median survival by 2–3 months	Several trials with similar results; we mention the most detailed one [a retrospective integration of results by Miller et al. (2012)]

We included clinical trials that are already completed independently of the fact if the results have been or not been published yet as well as ongoing searches for tag combinations of “Hsp70” with “neurodegeneration,” “neurodegenerative,” “AD,” “PD,” “HD,” “ALS,” “FTD” in all free and open accessible databases.

Multivector drugs, that affect different Hsp groups, attract a lot of interest. These are mainly geldanamycin and its derivatives, especially 17-AAG, 17-DMAG, IPI-504, and radicicol, which work as Hsp90-inhibitors and HSF-1-mediated Hsp70 inducers at the same time (Alam et al., 2017). Although, a predominance of Hsp70 or Hsp90 is still debatable (Rutledge et al., 2022). Several mechanisms of neuronal action can also be combined in a single medication. Thus, riluzole, which is known as anti-ALS basic treatment, is officially described by its producers as an antagonist of glutamate-associated excitotoxicity, but its effects are also provided by HSF-1 upregulation (Miller et al., 2012; Petri et al., 2023). Yang and coll. Report that riluzole may also enable Htt cleavage (Yang et al., 2008). Actually, there is no consensus on dynamics of riluzole action in literature.

Furthermore, some well-known medications may take part in the Hsp70-associated management of NDs. For instance, colchicine is actively tested together with riluzole due to its upregulation of HspB8 expression and blocking of TDP-43 accumulation (Mandrioli et al., 2019). Konturek et al. (2005) also studied pioglitazone and discovered its ability to improve Hsp70 content in the pancreas. However, a concomitant intake of pioglitazone and riluzole exhibits no clinical benefit in trials for ALS (Dupuis et al., 2012). In addition, fenofibrate causes a synchronous decrease in Hsp90 levels and elevation of Hsp70 levels, and cognitive dysfunction regresses in a rat model of dementia (Rizk et al., 2022). However, valproic acid enhances Hsp70 levels with no impact on Hsp90-machinery. Finally, carbenoxolone is efficient in proteasomal cleavage of aggregates *via* HSF-1 upregulation in PD rat models in the presence of rotenone (Thakur and Nehru, 2014).

Some Hsp70 enhancers with no clear mechanism found are suggested. For example, sleep deprivation-induced learning/memory impairment has been shown to recover after intracerebroventricular luteolin administration, while an increase in Hsp70 levels have accompanied the treatment (Rahimpour et al., 2023). Then, FLZ or phenlarmide alleviates motor dysfunction in animal PD models with an overexpression of Hsp70 (Kong et al., 2011; Bao et al., 2017). Additionally, some mechanisms are being developed with no pharmaceutical compounds proposed yet. Thus, cysteine string protein α (CSPα) phosphorylation by protein kinase C-γ promotes Hsp70 activity (Shirafuji et al., 2018).

Surprisingly, relative rare NDs tend to be more easily managed by Hsp70-recruiting drug options. So, arimoclomol (BRX-220), which is a low-molecular-weight hydroxylamine derivative Hsp70 enhancer, is tested in animal models of Fabry, Sandhoff, and Niemann-Pick type C (NPC) diseases (Kirkegaard et al., 2016). Mice of Gla−/− (Fabry), Hexb−/− (Sandhoff), and Npc1−/− (NPC) lines have shown a clinical improvement after arimoclomol intake. Hsp70 inducers/coinducers are generally convincing for ALS treatment (Kalmar et al., 2014; Kalmar and Greensmith, 2017), and it is especially true for arimoclomol (Phukan, 2010). Interestingly, that arimoclomol alone is effective but not efficient. It requires a preliminary heat shock induction to exhibit its potential (Kalmar et al., 2008).

Hsp70 inducers, mentioned above, are however very different by their biochemical features. This diversity has to be respected in practical implementation. For example, the compounds of geldanamycin group, in addition to their initial Hsp-related activity in cells, are able to prevent a transfer of receptors to steroid hormones into the nucleus, thereby providing a longer immune association (Czar et al., 1997). Geranyl geranylacetone acts *via* NF-κB-COX-2 axis to rule gene promoters, so cyclooxygenase-related adverse effects may be provoked (Nishida et al., 2007).

### 4.2. Recombinant Hsp70 and Hsp70-related genetic therapy

Exogenous recombinant Hsp70 (rHsp70ex) is a direct form of the chaperones that can be easily delivered into the body. It seems to be beneficial in NPC and AD models, particularly in familial AD (Kakimura et al., 2002; Mengel et al., 2021). For instance, rHsp70ex enhances memory and learning in AD models *in vivo* (Zatsepina et al., 2021). Then, cerebral and hippocampal cortex accumulates labeled rHsp70ex after intranasal administration (Yurinskaya et al., 2015). Further, mice with familial AD display declined Aβ levels and partial cognitive recovery after rHsp70ex administration (Bobkova et al., 2014; Evgen’ev et al., 2017). In addition, murine models of AD reflect a downregulation of neuroinflammatory markers after rHsp70ex in transcriptomic analysis of hippocampal neurons (Heppner et al., 2015; Yurinskaya et al., 2016). Moreover, neuroblasts proliferate and differentiate after intranasal rHSP70ex administration, perhaps due to the activation of cAMP 26 responsive element binding protein (CREB) cascade (Kwon et al., 2019). The treatment by rHsp70ex results in resistance to oxidative stress *via* mature endosomes and lysosomes with decreased apoptotic activity (Subrizi et al., 2015).

In a gene therapy study, rHsp70 has reached impaired dopaminergic neurons *via* an adenoviral vector with further decrease of neuronal loss (Dong et al., 2005). A similar effect is seen in Drosophila models for Hsp70 boosts both *via* gene therapy and induction by tanespimycin, a geldanamycin derivative (Zhang et al., 2016). Severity of PD signs has mitigated in sirtuin-1-transgenic mice due to the activation of HspA4 (Yang et al., 2022). However, HspA4 is not traditionally considered to belong to Hsp70 (Kaneko et al., 1997), although many controversial data appear recently (Shang et al., 2021; Abd El-Fadeal et al., 2023). A separate field of study includes epigenetic modifications of the chaperome, but it still remains weakly studied (Taldone et al., 2014).

### 4.3. Allosteric modulators of Hsp70

Allosteric modification represents an alternative way to control Hsp70 function. Surprisingly, practically all modulators can behave as Hsp inhibitors, although their overall impact depends on many factors. Avoiding concurrence, allosteric binding stays selective, supporting a comprehensive understanding of Hsp70-involving cascades (Ekimova and Plaksina, 2016; Gleixner et al., 2016; Ferraro et al., 2019). First, rhodacyanine-derived compounds are found to modify Hsp70 cooperation with its coactors in an allosteric site, inhibiting a reversive transformation of the chaperone’s molecule from ADP- into ATP-binding state and thereby improving protein holding (Li et al., 2016). The group includes MKT-077 and YM-01, which have been investigated for anti-AD activity as they may provoke Hsp70-mediated decline in pathological tau content *in vitro* (Abisambra et al., 2013; Martin et al., 2016). Furthermore, YM-08 is the next generation with a milder but also a less toxic action (Miyata et al., 2013), whereas its halogen-recruiting modification (JG-23) is even more chemically stable (Chang et al., 2021; Shao et al., 2021).

Allosteric Hsp70 regulation has been also proposed for some other pharmacological groups. For instance, phenothiazines such as methylene blue and azure C decrease total tau and phospho-tau levels due to the anti-ATP-ase activity, while the benefit of this effect is debatable (Martin et al., 2016; Lo Cascio and Kayed, 2018). Also, the neurotrophic compound J147 prevents synaptic loss in a transgenic murine AD model (Chen et al., 2011). However, J147 directly affects ATP-synthase, thereby triggering a bounce in intracellular calcium levels with a launch of 5′-adenosine monophosphate-activated protein kinase and mammalian target of rapamycin (AMPK/mTOR) pathway by kinase β of calcium/calmodulin-dependent protein kinase (CAMKK2) affecting mitochondrial metabolism (Goldberg et al., 2018). There is no proven Hsp70-involving action for J147.

Despite the new horizons of Hsp70 usage, current clinical trials focus mainly on Hsp90-modifiers or nonselective Hsp-controllers because of a better understanding of Hsp90 machinery (Thirstrup et al., 2016). Even more, pridopidine has been studied for HD treatment with a profound search on the impact of S1R chaperones and few data about Hsp engagement (Shenkman et al., 2021). However, chemical and biological modifications of Hsp70-involving pathways is a mighty impact spot in future management of NDs (Fontaine et al., 2016).

Perhaps, combining chemically different medications would provide additional options. So, Hsp70 overexpression strengthens in concomitant administration of Hsp90 inhibitors together with histone deacetylase inhibitors (Kuta et al., 2020). Some drugs are slightly effective with no strict pathway clear yet. That is, for example, the phenomenon of myricetin, bortezomib, and MG-132, counterparts that affect proteasome-mediated Hsp70 action (Joshi et al., 2019). Generally, these proteasome modulators (or JG substances) are studied for tumor cell management, as Grp78 and mtHsp70 machinery suffers in their presence (Cagala et al., 2020; Ferguson et al., 2022).

### 4.4. Nontrivial approaches for Hsp70 management in NDs

Curiously, physical methods might also contribute to Hsp70 machinery. For example, near-infrared irradiation tends to improve Hsp70 activity in the splenic and hepatic regions of mice (Escudero-Duch et al., 2023). The idea has also been developed for sauna heating inducing Hsp70 activation (Hunt et al., 2020). Hsp70 levels are generally elevated in increased physical activity (Kim et al., 2022). Phytotherapy also may represent a feasible approach. So, an extract of African walnut, or *Tetracarpidium conophorum*, is recently shown to improve the PD-like signs in rats, probably *via* Hsp70 modulation (Tokunbo et al., 2023). Neferine, derived from seeds of lotus plants, seems to increase neuronal tolerance to ischemia *via* Hsp70-induction machinery (Sengking et al., 2022).

## 5. Discussion

Molecular chaperones, mainly Hsp70 and Hsp90, play one of central biochemical roles providing structural volatility of proteins (Gupta et al., 2020). Hsp70 content should change rapidly to answer challenges of constantly transforming environment, and that is especially true for neurons as there are almost no proliferative or reconstructive machinery. In NDs, neuronal chaperome is usually impaired, so Hsp70 has a great potential to manage the pathology (Kim et al., 2021).

The challenge is that Hsp70 content in NDs shows no strict linear relation with the development of disease. For example, a decrease in plasma Hsp levels is observed at initial stages of AD and FTD, but changed into a recovery in moderate and severe cases (Chanteloup et al., 2019). It might be explained by gradual Hsp70 accumulation in the brain to repair neurons. However, the clinical implementation of this theoretical basis is more complex, because the state-of-the-art in Hsp70 studies for NDs still demonstrates a lot of white spots.

Nevertheless, Hsp70 have generally been found to be useful in neuronal functional and/or structural damage (Beretta and Shala, 2022). For instance, geranylgeranyl acetone induces Hsp70 expression in mice *via* HSF-1 with proven cognitive improvement (Sun et al., 2017). However, we still have no certain concept about principal differences between extra- and intracellular effects of Hsp70 in NDs. Theoretically, an intrinsic Hsp70 in NDs is responsible for aggregate cleavage, whereas an extrinsic Hsp70 joins immune interactions.

We consider Hsp70 to be a large field of studies in cellular and molecular biology for the upcoming years. For instance, we lack comprehensive research of Hsp70 potential in transgenic animals. Additionally, it seems that a well-known practice of physiological experiments with moderate exposure to high temperatures in humans and rats would obtain a second wind for testing the issue of Hsp70-modifying medications and transgenic Hsp70 for NDs. In a recent work, we have already tried an approach to synchronize physiological and morphological findings by laser speckle contrast imaging (Piavchenko et al., 2021; Konovalov et al., 2023).

## Author contributions

GP and AV: conceptualization. AV, VK, and AM: writing (original draft preparation). IM, AB, OB, GP, and AV: writing (review and editing). EK and AV: rendering, style design, and content presentation. All authors have read and agreed to the published version of the manuscript.

## Funding

This study was supported by the Russian Science Foundation (project no. 23–25-00448, https://www.rscf.ru/project/23-25-00448/). Our review launches a series of works according to the grant in study of neuroimmunological impacts of extra- and intracellular Hsp70 in murine models of neurodegeneration.

## Conflict of interest

The authors declare that the research was conducted in the absence of any commercial or financial relationships that could be construed as a potential conflict of interest.

## Publisher’s note

All claims expressed in this article are solely those of the authors and do not necessarily represent those of their affiliated organizations, or those of the publisher, the editors and the reviewers. Any product that may be evaluated in this article, or claim that may be made by its manufacturer, is not guaranteed or endorsed by the publisher.
